# Epidemiology of nasopharyngeal carriage of respiratory bacterial pathogens in children and adults: cross-sectional surveys in a population with high rates of pneumococcal disease

**DOI:** 10.1186/1471-2334-10-304

**Published:** 2010-10-23

**Authors:** Grant A Mackenzie, Amanda J Leach, Jonathan R Carapetis, Janelle Fisher, Peter S Morris

**Affiliations:** 1 Child Health Division, Menzies School of Health Research, Darwin, Australia; 2 Flinders University, School of Medicine, Adelaide, Australia; 3 Charles Darwin University, Darwin, Australia; 4 NT Clinical School, Flinders University, Darwin, Australia

## Abstract

**Background:**

To determine the prevalence of carriage of respiratory bacterial pathogens, and the risk factors for and serotype distribution of pneumococcal carriage in an Australian Aboriginal population.

**Methods:**

Surveys of nasopharyngeal carriage of *Streptococcus pneumoniae*, non-typeable *Haemophilus influenzae*, and *Moraxella catarrhalis *were conducted among adults (≥16 years) and children (2 to 15 years) in four rural communities in 2002 and 2004. Infant seven-valent pneumococcal conjugate vaccine (7PCV) with booster 23-valent pneumococcal polysaccharide vaccine was introduced in 2001. Standard microbiological methods were used.

**Results:**

At the time of the 2002 survey, 94% of eligible children had received catch-up pneumococcal vaccination. 324 adults (538 examinations) and 218 children (350 examinations) were enrolled. Pneumococcal carriage prevalence was 26% (95% CI, 22-30) among adults and 67% (95% CI, 62-72) among children. Carriage of non-typeable *H. influenzae *among adults and children was 23% (95% CI, 19-27) and 57% (95% CI, 52-63) respectively and for *M. catarrhalis*, 17% (95% CI, 14-21) and 74% (95% CI, 69-78) respectively. Adult pneumococcal carriage was associated with increasing age (p = 0.0005 test of trend), concurrent carriage of non-typeable *H. influenzae *(Odds ratio [OR] 6.74; 95% CI, 4.06-11.2) or *M. catarrhalis *(OR 3.27; 95% CI, 1.97-5.45), male sex (OR 2.21; 95% CI, 1.31-3.73), rhinorrhoea (OR 1.66; 95% CI, 1.05-2.64), and frequent exposure to outside fires (OR 6.89; 95% CI, 1.87-25.4). Among children, pneumococcal carriage was associated with decreasing age (p < 0.0001 test of trend), and carriage of non-typeable *H. influenzae *(OR 9.34; 95% CI, 4.71-18.5) or *M. catarrhalis *(OR 2.67; 95% CI, 1.34-5.33). Excluding an outbreak of serotype 1 in children, the percentages of serotypes included in 7, 10, and 13PCV were 23%, 23%, and 29% (adults) and 22%, 24%, and 40% (2-15 years). Dominance of serotype 16F, and persistent 19F and 6B carriage three years after initiation of 7PCV is noteworthy.

**Conclusions:**

Population-based carriage of *S. pneumoniae*, non-typeable *H. influenzae*, and *M. catarrhalis *was high in this Australian Aboriginal population. Reducing smoke exposure may reduce pneumococcal carriage. The indirect effects of 10 or 13PCV, above those of 7PCV, among adults in this population may be limited.

## Background

*Streptococcus pneumoniae *is responsible for an estimated 14.5 million episodes of serious disease and 826,000 deaths annually among children aged 1 to 59 months [1]. The burden of pneumococcal disease in older age groups is unknown. *Streptococcus pneumoniae *is the most common cause of pneumonia [2,3] while both non-typeable *Haemophilus influenzae *(indicated subsequently as *H. influenzae*) and *Moraxella catarrhalis *are associated with exacerbations of chronic obstructive airways disease [4,5]. Among Australia's Northern Territory (NT) Aboriginal population, respiratory causes are responsible for 18% of deaths [6]. Aboriginal adults and older children also experience high rates of invasive pneumococcal disease (IPD) [7].

Respiratory infection in individuals is related to episodes of pneumococcal carriage acquisition [8,9]. Pneumococcal conjugate vaccines (PCV) reduce carriage of vaccine serotypes (VT) with increased carriage of non-vaccine serotypes [10,11]. The importance of the impact of PCV on carriage has been highlighted by the large indirect effects observed following introduction of 7-valent PCV (7PCV) in the United States [12]. However, the characteristics of population-level pneumococcal carriage epidemiology which influence the indirect effects of PCV are not well described. Whether such indirect effects can be expected in populations with different pneumococcal carriage epidemiology is unknown.

Data concerning nasopharyngeal carriage of *S. pneumoniae*, *H. influenzae*, and *M. catarrhalis *in high-risk populations, such as Aboriginal adults and older children, are limited. The NT of Australia introduced 7PCV and 23-valent pneumococcal polysaccharide vaccine (23PPV) for Aboriginal children in late 2001. Despite high levels of vaccination coverage, a study describing the indirect effects of universal pneumococcal vaccination in the four Aboriginal communities involved in this study documented the same VT carriage prevalence in 2002 and 2004 [13]. Further aims of this study were to determine the age-specific prevalence of respiratory bacterial carriage in children and adults, as well as the risk factors for and serotype distribution of pneumococcal carriage among those aged 2 years and over.

## Methods

### Study population

We performed two surveys in the four main communities (> 90% of the total population) on the Tiwi Islands, 70 km north of Darwin, off the coast of the NT. In 2002, the population was 2,204 and 92% of individuals identified as Aboriginal. NT immunization policy recommended 23PPV for Aboriginal adults > 15 years of age. A 7PCV catch-up campaign for those < 2 years of age began in August 2001 along with three doses of 7PCV at 2, 4, and 6 months of age and booster 23PPV at 18 months of age.

### Procedures

Participants were enrolled in 2002 (August - November) and these individuals were followed up, along with others in 2004 (March - May). The 2002 and 2004 surveys were conducted during dry and wet seasons respectively. Eligibility criteria were: permanent residence, Aboriginal ethnicity, and age ≥2 years. Purposive sampling aimed to obtain equal numbers of 'typical' males and females in each of two groups; adults (≥16 years of age) and children (≥2 and < 16 years of age). To obtain more precise estimates of prevalence among adults with lower carriage than children, sampling aimed to enrol 1.5 times the number of adults than children. We visited a range of work places, public locations, schools and private residences.

Written informed consent was obtained after participants read the study information or had it explained to them in English or Tiwi. When consent was discussed with adults, consent was also sought for enrolment of their children. The study was conducted in accordance with the Declaration of Helsinki and was approved by the Tiwi Health Board and the NT Department of Health and Community Services, Human Research Ethics Committee.

Demographic and risk factor data, as well as information from community clinic records, were recorded on standard forms. Dates of pneumococcal and influenza vaccination were extracted from clinic records, the community clinic and NT immunisation databases.

### Microbiological methods

Nasopharyngeal swabs and culture used methods similar to those proposed by WHO [14]. Methodological variations were: swab placement for 10 rather than 5 seconds and rotation 360° rather than 180°. Aluminium-shafted, cotton-tipped swabs (Disposable Products, Australia) were introduced horizontally into the nasal cavity 8-10 cm or until resistance was encountered. If the first swab was not tolerated a second swab was performed or nasal secretions were collected from a tissue used to blow the nose [15]. Swabs were placed immediately into skimmed milk glucose glycerol broth and stored at -20°C for transport to the laboratory for storage at -70°C. A 10 μL loop of broth was streaked on bacitracin containing horse blood agar and chocolate agar (Oxoid, Australia). Plates were incubated overnight at 37°C in 5% CO_2_. Isolation of *S. pneumoniae *was confirmed by colony morphology, optochin sensitivity and serotyping by the Quellung reaction (Statens Serum Institut, Denmark), *H. influenzae *by dependence on X and V factors, and *M. catarrhalis *by colony morphology, Gram stain and oxidase production.

### Statistical analysis

Data from 2002 and 2004 were aggregated for analysis of risk factors based on the following criteria: a) carriage prevalence in 2002 and 2004 not significantly different, b) concordant direction of association in univariate analyses, and c) univariate associations showing p ≤ 0.20. Multiple logistic regression was used for risk factor analysis. Adjustment for correlation between individual pairs of observations in the two surveys was made by including participant identity as a clustering variable in logistic models. Effect modification between variables was evaluated.

Given carriage of 40% in Aboriginal mothers in these communities, the primary risk factor for analysis was sex. A 30% prevalence in men was assumed to be clinically significantly different from women. A sample size of 376 males and 376 females was required to detect the difference at the 5% significance level with power of 0.8.

## Results

At the time of the 2002 survey, 94% of eligible children had received pneumococcal catch-up vaccination and the mean age at the third dose was 6.7 months (range 4.2 - 13.6). In 2004, all eligible children had received three doses of 7PCV with 33% receiving the third dose greater than 7 months of age. The proportion of participants < 5 years of age, who had received 7PCV in 2002 and 2004, was 30% and 73% respectively. In 2002, 87% of participants ≥15 years had received 23PPV.

**Figure 1:**
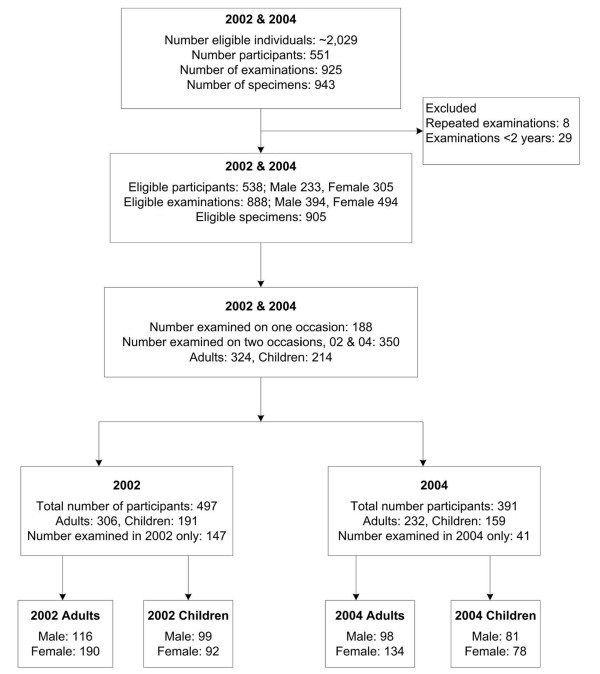
**Enrolment and follow-up profile**. Note: Number of eligible adults (324) and children (218) is not equal to the total number of eligible participants (538) as four participants were categorized as children in 2002 and then categorized as adults in 2004.

A total of 943 specimens were collected from 925 examinations of 551 participants. After exclusions, 905 specimens from 888 examinations of 538 participants remained for analysis (Figure 1). Of all examinations, 60.6% were in adults (> 16 years) and 44.4% were in males. Males comprised a smaller proportion of examinations among adults (Figure 1).

The prevalence of pneumococcal carriage in all examinations was 42.3% (376/888). Carriage prevalence among adults was 28.4% in 2002 and 22.8% in 2004, odds ratio (OR) 0.75 (95% CI, 0.54-1.02). Carriage among children in 2002 was 69.6% and 64.8% in 2004 (OR 0.80; 95% CI, 0.54-1.19). *H. influenzae *carriage in all examinations was 36.5% (324/888), while carriage prevalence among adults was 24.8% in 2002 and 20.3% in 2004; carriage prevalence in children was 61.3% in 2002 and 52.8% in 2004. *M. catarrhalis *carriage in all examinations was 39.4% (350/888), 15.7% among adults in 2002 and 19.0% in 2004 with carriage among children of 74.3% in 2002 and 73.0% in 2004 (Table 1).

**Table 1:** Prevalence of *H. influenzae, M. catarrhalis *and *S. pneumoniae *carriage by age group (aggregate data from 2002 and 2004)

Age group (yrs)	*H. influenzae *carriage n/N (%)	*M. catarrhalis *carriage n/N (%)	*S. pneumoniae *carriage n/N (%)	*Odds ratio *S. pneumoniae *carriage (95% CI)
Adults				
				
16-24	26/133 (19.6)	14/133 (10.5)	23/133 (17.3)	1
25-34	35/135 (25.9)	29/135 (21.5)	30/135 (22.2)	1.37 (0.68-2.74)
35-44	23/120 (19.2)	21/120 (17.5)	37/120 (30.8)	2.13 (1.09-4.15)
45-54	22/95 (23)	15/95 (16)	29/95 (31)	2.10 (1.04-4.26)
≥55	17/55 (31)	13/55 (24)	21/55 (38)	2.95 (1.30-6.69)
Total	123/538 (22.9)	92/538 (17.1)	140/538 (26.0)	
				
Children				

2-4	70/102 (68.6)	91/102 (89.2)	84/102 (82.4)	1
5-8	84/132 (63.6)	104/132 (78.8)	96/132 (72.7)	0.57 (0.29-1.11)
9-12	37/93 (40)	53/93 (57)	48/93 (52)	0.23 (0.12-0.45)
13-15	10/23 (44)	10/23 (43)	7/23 (30)	0.09 (0.03-0.27)
Total	201/350 (57.4)	258/350 (73.7)	235/350 (67.1)	

* Odds ratios and confidence intervals calculated using logistic regression and adjusted for repeated measures on an individual. Score tests for trends of odds across adult and child age groups: *H. influenzae - *Adults, p = 0.28; Children, p = 0.0001. *M. catarrhalis - *Adults, p = 0.12; Children, p < 0.0001. *S. pneumoniae - *Adults, p = 0.0005; Children, p < 0.0001.

**Figure 2:**
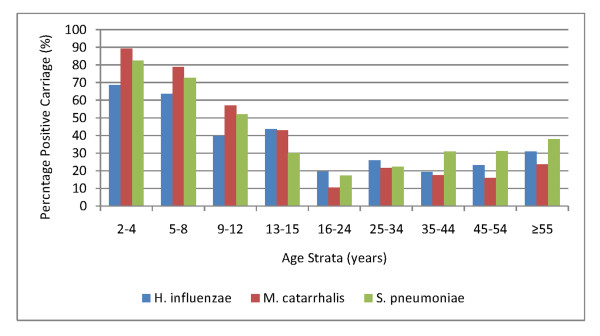
**Carriage of *H. influenzae*, *M. catarrhalis*, and *S. pneumoniae *by age group (in years) in the two surveys combined**.

Pneumococcal carriage was greatest in young children and older age categories (Figure 2). Among adults there was a trend of increasing carriage with increasing age (Table 1). The trend among children was even greater but inverse, pneumococcal carriage decreased greatly with increasing age (Table 1). Carriage of *H. influenzae *and *M. catarrhalis *showed no pattern over adult age groups but carriage fell with increasing age of children (Figure 2).

Among adults, univariate analyses showed pneumococcal carriage was associated with male sex, chest infection in the previous month, recent runny nose, 1-2 household occupants < 5 years of age, frequency sitting at an outside fire, a young child as the closest personal contact and concurrent carriage of *H. influenzae *or *M. catarrhalis *(Table 2). Among children, carriage was associated with recent runny nose, the number of household occupants < 5 years of age, the number of bedroom occupants < 5 years of age, and concurrent carriage of *H. influenzae *and *M. catarrhalis *(Table 2).

**Table 2:** Adults and children, univariate associations between explanatory variables and pneumococcal carriage (aggregate data for 2002 and 2004)

Variable		Adults				Children		
		
		Positive carriage (%)	Odds ratio	p-value		Positive carriage (%)	Odds ratio	p-value
Sex	Female	72/324 (22)	1			n/a	n/a	
	Male	68/214 (32)	1.63 (1.04-2.56)	0.03				
								
Chest infection in the previous month	No	67/329 (20)	1			n/a	n/a	
	Yes	70/200 (35)	2.11 (1.40-3.18)	< 0.01				
								
Runny nose in the previous week	No	60/306 (20)	1			76/142 (54)	1	
	Yes	71/214 (33)	2.04 (1.37-3.02)	< 0.01		147/187 (79)	3.19 (1.97-5.17)	< 0.01
								
Number household occupants	0	92/306 (30)	1			59/103 (57)	1	
	1-2	37/187 (20)	0.57 (0.37-0.89)	0.01		136/191 (71)	1.84 (1.10-3.08)	0.02
< 5 years	≥3	10/39 (26)	0.80 (0.36-1.77)	0.58		38/49 (78)	2.58 (1.14-5.83)	0.02
								
Number bedroom occupants	0	n/a	n/a			142/224 (63)	1	
	1					27/40 (68)	1.35 (0.63-2.90)	0.44
< 5 years	≥2					67/86 (78)	2.12 (1.18-3.81)	0.01
								
Sits at an outside fire						n/a	n/a	
Never/monthly		35/172 (20)	1					
Most days		96/347 (28)	1.50 (0.99-2.26)	0.05				
Every day		7/11 (64)	6.9 (1.9-24.5)	< 0.01				
								
Daytime location		n/a	n/a		School	167/263 (64)	1	
					Creche	14/14 (100)	n/a	
					Home	22/31 (71)	1.41 (0.64-3.07)	0.39
					With carer	32/36 (89)	4.60 (1.60-13.2)	< 0.01
								
Closest personal contact								
Adult		118/403 (29)	1		School	170/268 (63)	1	
School child		4/25 (16)	0.46 (0.15-1.41)	0.18	Creche	20/22 (91)	5.76 (1.29-25.8)	0.02
Creche child		3/14 (21)	0.66 (0.17-2.50)	0.54	Mother	43/50 (86)	3.54 (1.55-8.1)	< 0.01
Young child		14/88 (16)	0.46 (0.23-0.90)	0.02				
								
*H. influenzae *detected	No	68/415 (16)	1			57/149 (38)	1	
	Yes	72/123 (59)	7.20 (4.59-11.3)	< 0.01		178/201 (89)	12.49 (6.96-22.4)	< 0.01
								
*M. catarrhalis *detected	No	94/446 (21)	1			35/92 (38)	1	
	Yes	46/92 (52)	3.74 (2.36-5.95)	< 0.01		200/258 (78)	5.62 (3.21-9.81)	< 0.01

Note: Odds ratios and confidence intervals calculated by logistic regression and adjusted for repeated measures. n/a, not applicable. Adult risk factors excluded from aggregation of 2002 and 2004 data: antibiotic prescription in the previous month, number of bedroom occupants, diagnosis of rheumatic heart disease or chronic lung disease (data not shown), current smoker, fire outside the house, 23PPV in the previous 2 years, number of bedroom occupants < 5 years of age, bedroom occupant includes a child, employed, record of proteinuria or diabetes, influenza vaccination in the previous 2 years, record of excessive alcohol use. Risk factors included in aggregate data: sex, chest infection in the previous month, runny nose in the previous week, number of household occupants < 5 years of age, closest personal contact (young child versus adult), and concurrent carriage of *H. influenzae *or *M. catarrhalis*. Frequency sitting at an outside fire was included in aggregate data as p = 0.003 for one category in 2002 but no observations in that category in 2004. Among children, risk factors excluded from aggregation: antibiotic prescription in the previous month, fire outside the house, number of household occupants, number of bedroom occupants, and bedroom occupant includes an adult. Risk factors included in aggregate data: runny nose in the previous week, number of household occupants < 5 years of age, number of bedroom occupants < 5 years of age, daytime location, closest personal contact (mother versus school children), and concurrent carriage of *H. influenzae *or *M. catarrhalis*.

Multivariate analysis showed independent risk factors (10% significance level) for pneumococcal carriage among adults were: increasing age, male sex, chest infection in the previous month, runny nose in the previous week, frequency of sitting at an outside fire and concurrent carriage of *H. influenzae or M. catarrhalis *(Table 3). Among children, independent risk factors for carriage were decreasing age, runny nose in the previous week, and concurrent carriage of *H. influenzae *or *M. catarrhalis *(Table 3).

**Table 3:** Multivariate model of risk factors for pneumococcal carriage among adults and children

Risk factor	Adults		Children	
	
	Category	Odds Ratio (95% CI)	Category	Odds Ratio (95% CI)
Age group	16-24 yrs	1	2-4 yrs	1
	25-34 yrs	1.13 (0.52-2.49)	5-8 yrs	0.65 (0.28-1.54)
	35-44 yrs	2.04 (0.92-4.54)	9-12 yrs	0.43 (0.17-1.08)
	45-54 yrs	1.90 (0.83-4.34)	13-15 yrs	0.13 (0.03-0.56)
	≥55 yrs	2.19 (0.88-5.50)		
				
Sex	Female	1	n/a	
	Male	2.21 (1.31-3.73)		
				
Chest infection in previous month	No	1	n/a	
	Yes	1.62 (0.98-2.67)		
				
Runny nose in previous week	No	1	No	1
	Yes	1.66 (1.05-2.64)	Yes	1.80 (0.98-3.29)
				
Frequency sitting at an outside fire	Never/monthly	1	n/a	
	Most days/weekly	1.23 (0.74-2.05)		
	Every day	6.89 (1.87-25.4)		
				
*H. influenzae *detected	No	1	No	1
	Yes	6.74 (4.06-11.2)	Yes	9.34 (4.71-18.5)
				
*M. catarrhalis *detected	No	1	No	1
	Yes	3.27 (1.97-5.45)	Yes	2.67 (1.34-5.33)

Note: Odds ratios and confidence intervals calculated by logistic regression adjusting for repeated measures on an individual.

There was no effect modification of *H. influenzae *or *M. catarrhalis *carriage, with risk of pneumococcal carriage, nor effect modification of pneumococcal carriage and carriage of *H. influenzae *or *M. catarrhalis*.

**Figure 3:**
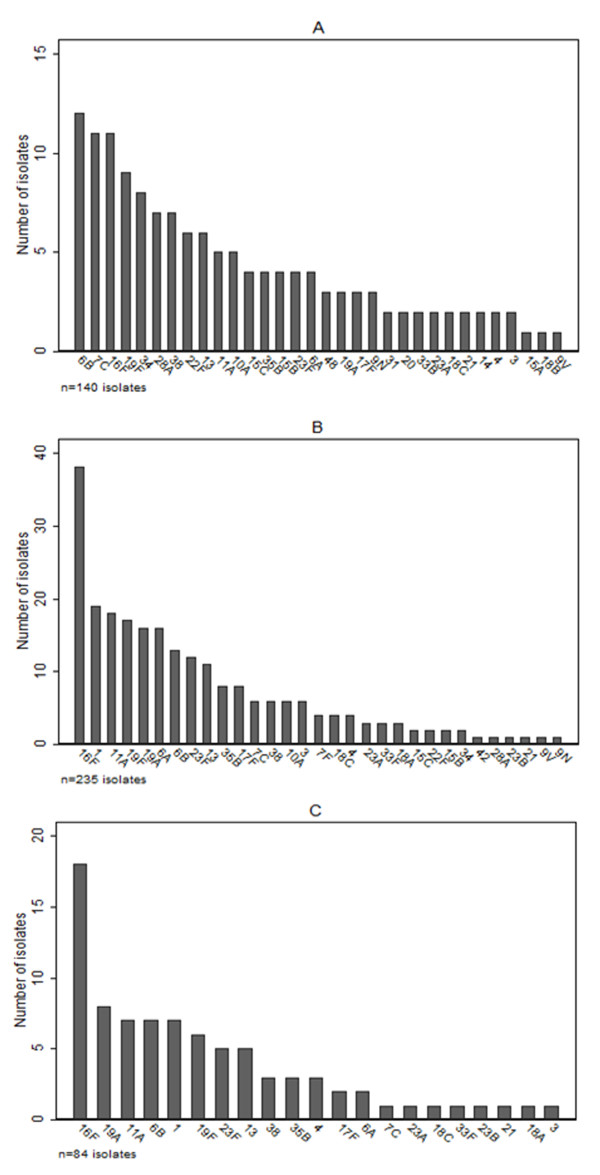
**Pneumococcal carriage serotype distributions in the two surveys combined: A) Adults, B) Age < 15 years, and C) Age < 5 years**.

Thirty-nine pneumococcal serotypes were identified. Among adults the predominant types were 6B, 7C, 16F, 19F, and 34 (Figure 3A). Among those < 15 years, serotypes 16F, 1, 11A, 19F, and 6A predominated (Figure 3B), while among those < 5 years, serotypes 16F, 19A, 6B, 1, and 11A predominated (Figure 3C). An epidemic of serotype 1 carriage was evident in 2002 [16]. Serotype 1 carriage was not detected in 2004. Excluding the outbreak of serotype 1, the percentages of serotypes included in the 7, 10, and 13-valent PCV were 23%, 23%, and 29% (adults), 22%, 24%, and 40% (< 15 years), and 26%, 26%, and 40% (< 5 years) respectively. Dominance of serotype 16F, and persistent 19F and 6B carriage (particularly among adults) is noteworthy (Figure 3).

## Discussion

This is the first population-based study of respiratory bacterial carriage prevalence and risk factors among Australian Aboriginal adults and older children. Pneumococcal carriage prevalence was 67.4% in children age 2-15 years and 26.0% in adults. The prevalence of *H. influenzae *carriage was 57.4% in 2-15 year old children and 22.9% in adults. Of the three pathogens, *M. catarrhalis *was the most prevalent in children (73.7%), and the least prevalent in adults (17.1%). Pneumococcal carriage among adults was associated with increasing age, male sex, recent chest infection, recent rhinorrhoea, frequency of sitting at an outside fire, and concurrent carriage of *H. influenzae *or *M. catarrhalis*. Among older children, pneumococcal carriage was strongly associated with younger age and concurrent carriage of *H. influenzae *or *M.catarrhalis *and less strongly associated with recent rhinorrhoea. A large number of pneumococcal serotypes circulate in this population. Higher valency PCVs would cover a greater proportion of serotypes in children than adults.

Compared to other population-based studies of pneumococcal carriage, the 42% overall prevalence in this population (mean age 25 years) was midway between that observed among Gambian villagers (65%, median age 15 years) [17] and in a Brazilian slum (36%, median age 16 years) [18] or Kilifi, Kenya (20%, mean age 9 years) [19]. Lower carriage (> 2 years) has been reported in the UK (16%) [20] and Taiwan (10%) [21].

Pneumococcal carriage among children (67%) in this population (mean age 8 years) was comparable to 82% in 5 to 14 year old Gambians [17] and greater than among 5 to 19 year olds in Kilifi (25%), 5 to 17 year old Brazilian slum residents (45%), and Brazilian adolescents (10%) [22]. Pneumococcal carriage among adults in this population (26%) was lower than in Gambia (50%), similar to a Brazilian slum (20%), and greater than in Kilifi (3%), the UK (7%), or Taiwan (0%). An earlier study from Central Australia reported pneumococcal carriage in 34% of a selected group of Aboriginal adults [23]. The differences in prevalence noted above are difficult to explain due to a lack of data on methodology and comparable risk factors.

*H. influenzae *carriage in children (63%) was greater than that reported in north Indian primary school children (42%, 5-10 years) [24] or Kilifi (22%, 3-9 years) [19]. Carriage of *M. catarrhalis *(74% children, 17% adults) was greater than that reported in previous studies of healthy children (51%) and adults (5%) in Belgium [25] and hospitalized children (7%, 4-15 years) and adults (1%) in Denmark [26].

The decreasing pneumococcal carriage with increasing age of children has been described in other studies, albeit with varied patterns in different populations [17-21]. Of note, the studies cited here have been conducted in populations before introduction of 7PCV. Carriage prevalence of *H. influenzae *and *M. catarrhalis *among children also fell with increasing age.

Interestingly, the increased pneumococcal carriage with increasing adult age (38% ≥55 years versus 20% among 16 to 34 year olds) that we observed was not seen in The Gambia or Kilifi. The reason for increased carriage in older adults is not clear. It is unlikely that the trend of increasing pneumococcal carriage is due to chance (test for trend 2002, p = 0.05; 2004, p = 0.003; overall, p = 0.0005). Patterns of social interaction, particularly exposure to children, are usually less intense in older adults but may vary in different settings. Biological (e.g. immunosenescence), environmental (e.g. smoke exposure, stress), and chronic disease factors may also be important.

Odds of pneumococcal carriage were substantially increased if *H. influenzae *or *M. catarrhalis *were also detected. The Kilifi study reported a similar magnitude of association between pneumococcal carriage and concurrent carriage of *H. influenzae *[19]. The association with detection of other pathogens may reflect either facilitation of pneumococcal carriage by *H. influenzae *or *M. catarrhalis*, or simply non-species specific risk of nasopharyngeal carriage.

Our data, as well as contemporary [19,27] and historical [28] studies, describe increased pneumococcal carriage associated with upper respiratory infections. Our data also indicate an association of recent chest infection with increased pneumococcal carriage in adults. Episodes of pneumonia are associated with increased pneumococcal carriage [29,30].

We found 2.2 times the odds of pneumococcal carriage among adult males compared to females. An association of carriage with gender has not been reported in most studies [17,19,27,28], an exception being one study of adolescents [22]. Some of the factors potentially associated with both gender and carriage, such as contact with children, chronic illness, smoking or sitting at an outside fire, were included in multivariate analyses.

Pneumococcal carriage and respiratory infection have been associated with exposure to air pollution of different types [22,31]. We found the frequency of sitting at an outside fire increased the risk of pneumococcal carriage. Sitting and cooking at outside fires is common in rural Aboriginal communities. Thus, our data strengthen the growing body of evidence linking smoke exposure to increased risk of respiratory infection.

The large number of pneumococcal carriage serotypes which were detected suggests that pneumococcal transmission is substantial in this population. Our data suggest that the indirect effects of higher valency PCV may be greater than 7PCV (particularly for young infants who acquire *S. pneumoniae *from their siblings), with a more limited incremental effect among adults.

Our study has a number of limitations. Our sample collection methods underestimate carriage prevalence. Non-probability sampling may have introduced selection bias. Sampling aimed to identify equal numbers of typical adult men and women, with children numbering two thirds that of adults. However, sampling was biased towards adult women, and given lower carriage in women, adult carriage may be underestimated. Analyses of risk factors need to be interpreted in the light of possible sampling bias. Despite non-probability sampling, valid prevalence estimates are likely to fall within the given confidence intervals. Finally, this study is limited to a three year period following the introduction of 7PCV. Although carriage of serotypes 19F and 6B in 2004 remained substantial, it may have fallen in subsequent years. Another carriage study across the NT documented little change in childhood carriage of 19F and 23F between 2003 and 2005 but a reduction in carriage of 6B was observed [32].

The implications for public health relate to the introduction of PCV and prevention of pneumococcal transmission and disease. High overall carriage prevalence indicates a large proportion of the population contributing to pneumococcal transmission, which is consistent with the high rates of IPD observed among Aboriginal adults [7]. Such a reservoir of potentially transmitting individuals may maintain circulation of pneumococcal serotypes after introduction of PCV and limit the magnitude, and increase the time to development, of indirect effects. High rates of bacterial carriage also suggest potential for replacement disease following reduction of VT carriage. Ongoing pneumococcal transmission among Aboriginal adults may explain data showing no reduction in rates of IPD in Aboriginal adults following introduction of 7PCV despite a 30% to 45% reduction in IPD among all Australian adults following 7PCV introduction [33].

## Conclusion

Nasopharyngeal carriage of *S. pneumonia, H. influenzae*, and *M. catarrhalis *is high in this population. These data indicate that frequent exposure to outside fires and prevalent respiratory viral and bacterial infections all play a role in maintaining high rates of pneumococcal carriage. Interventions which reduce smoke exposure may reduce pneumococcal carriage. Among adults, low serotype coverage of higher valency PCV suggests that increased herd protection above that of 7PCV may be limited, although higher coverage among children, particularly during a serotype 1 outbreak, suggests that increased herd protection will be afforded to young infants. Ongoing carriage of 7PCV serotypes 19F and 6B is noteworthy, although our data are limited to a three year period following introduction of 7PCV.

## Declaration of competing interests

Peter Morris and Amanda Leach have received research funding from Wyeth Vaccines and GlaxoSmithKline and Peter Morris has acted as a consultant for GlaxoSmithKline. The other authors declare that they have no competing interests.

## Authors' contributions

GM conceived of the study, and participated in its design, coordination, data collection, and manuscript preparation. JF participated in coordination of the study, data collection and manuscript preparation. AL, JC, and PM participated in the design of the study and manuscript preparation. All authors read and approved the final manuscript.

## Note

This work was funded by Wyeth Australia, the National Health & Medical Research Council of Australia and Menzies School of Health Research. This work was undertaken at Menzies School of Health Research, PO Box 41096, Casuarina, Darwin, NT, Australia. The sponsor had no role in study design, data collection, analysis and interpretation, the writing of the report, or the decision to submit the manuscript for publication. Peter Morris and Amanda Leach have received research funding from Wyeth and GlaxoSmithKline and Peter Morris has acted as a consultant for GlaxoSmithKline. Grant Mackenzie is now affiliated with the Medical Research Council (UK) The Gambia.

## Pre-publication history

The pre-publication history for this paper can be accessed here:

http://www.biomedcentral.com/1471-2334/10/304/prepub
